# Looking back and looking forward at Janelia

**DOI:** 10.7554/eLife.44826

**Published:** 2019-02-07

**Authors:** Gerald M Rubin, Erin K O'Shea

**Affiliations:** 1 Janelia Research CampusHoward Hughes Medical InstituteAshburnUnited States; 2 Howard Hughes Medical InstituteChevy ChaseUnited States

**Keywords:** Janelia Research Campus, careers in science, science policy, organization of research, *D. melanogaster*, Mouse, Rat, Virus, Zebrafish

## Abstract

Starting a new research campus is a leap of faith. Only later, in the full measure of time, is it possible to take stock of what has worked and what could have been done better or differently. The Janelia Research Campus opened its doors 12 years ago. What has it achieved? What has it taught us? And where does Janelia go from here?

## Looking back

By the early 2000s, the interdisciplinary nature of many of the most interesting research questions in the life sciences was widely acknowledged. However, basic scientists were routinely hitting technical barriers that blocked progress, despite the ample resources available at many academic research labs. The Howard Hughes Medical Institute (HHMI), reasoning that efforts to break through these barriers were often impeded by certain features of the academic world, decided to create a research environment complementary to, and synergistic with, that offered by universities and other research institutions (Rubin, 2006).

**Figure 1.**
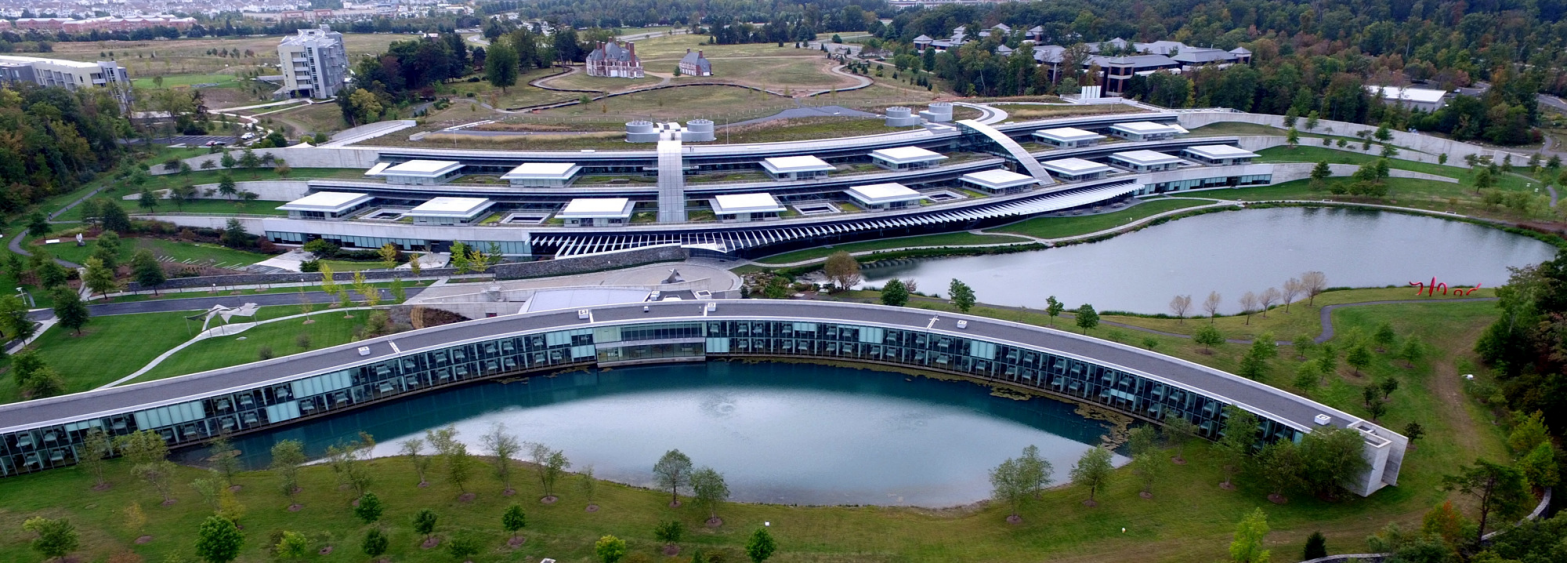
A view of the Janelia Research Campus in 2015. The building in the foreground is the 96-room guest house, primarily used to house conference participants and other short-term visitors. Above the guest house is the main research building, a three-story structure terraced into the side of a hill. The brick farm and carriage houses belonging to the original Janelia Farm can be seen near the top center. To the left are 60- and 80-unit apartment buildings that, along with 21 studio apartments and 34 multi-bedroom townhouses (not shown in this view), provide housing for more than one-quarter of those working on campus. An additional 100-unit apartment building and six single-family residences are planned. The Potomac River is approximately 300 meters to the North (downward). The campus covers an area of 281 acres plus a 408-acre island, Selden Island, located in the Potomac River and connected by a bridge. Photograph credit: Anthony Leonardo/HHMI.

That idea became HHMI’s Janelia Research Campus (Figure 1), a state-of-the-art research campus and community of more than 350 scientists, split between individual research labs, project teams and shared scientific support groups. Located on the banks of the Potomac River 55 kilometers upstream from Washington, DC, Janelia currently accounts for just under 15% of HHMI’s annual operating budget. Funding 300 HHMI investigators, who are typically group leaders working at universities and research institutions, was and continues to be the largest item in the HHMI budget.

Janelia’s original mission statement was "to identify important biomedical problems for which future progress requires technological innovation and then foster the establishment of integrated teams of biologists and tool-builders who seek to break through existing barriers." To accomplish this goal, HHMI first and foremost focused on culture. In particular, the new research campus would have organizational and reward structures very different from those found in academia. Research groups would be small and internally funded, group leaders would be expected to stay actively involved in bench research, and collaboration on long-term, multi-disciplinary research would be encouraged.

Only after these core cultural characteristics were firmly embedded in the plan for Janelia and construction of the campus was underway did HHMI organize a series of planning workshops to determine Janelia’s initial research areas. Two emerging areas were selected: i) understanding how information is stored and processed by neuronal circuits; and ii) developing novel imaging methods and computational tools for image analysis. These areas were judged to be under-supported relative to their potential impact and well suited to benefit from Janelia’s atypical research environment.

Of course, the fields of neuroscience and microscopy were around long before Janelia opened its doors. Yet, over 12 years, Janelia has created new opportunities and research directions in both of these areas. We believe this success stems not only from our choice of research problems, but also from the creation of a unique scientific culture that rewards interdisciplinary and collaborative work, and is supported by talent development and appointment structures different from those found in academic research programs.

### Janelia’s scientific culture

We see the following operational and philosophical features as central to Janelia’s scientific culture and, collectively, they distinguish Janelia from other research institutions:

i) Limiting lab size in order to enable group leaders to stay active in the direct conduct of research and to promote collaboration between groups.

ii) Providing institutional funding for all research. We collaborate with other organizations but do not accept government grants.

iii) Encouraging a focus on identifying important problems and pursuing answers with long-term commitment rather than emphasizing short-term deliverables. Focusing on a small number of challenging problems has promoted collaboration and allowed small labs to thrive.

iv) Providing long-term support for the development of widely useful, validated reagents, instrumentation, techniques, and conceptual approaches, drawing broadly from different fields (optical physics, chemistry, genetics, computer science and engineering). An institutional philosophy of disseminating information and tools to the broader community allows resident tool-builders to have an impact far beyond Janelia’s walls. For example: four of our novel microscopes have been, or are in the process of being, commercialized; we have granted over 270 research or open-source hardware licenses as well as provided detailed plans to individuals who want to build their own copies of these and other instruments; over 5,600 aliquots of our novel fluorescent dyes have been provided to more than 900 laboratories, and commercialized variants are available from multiple sources; our GAL4 drivers for targeted gene expression in Drosophila have been distributed to 1,500 laboratories by the Bloomington Drosophila Stock Center, and plasmid constructs for use with them have been distributed over 2,700 times by Addgene.

v) Creating unparalleled scientific support through shared facilities run by expert staff. These facilities expand the technical range of small labs by providing services such as instrument design and fabrication, software development, transgenic animals, animal care, cell culture, histology, electron microscopy, and molecular biology to all Janelia researchers.

vi) Fostering collaboration and investment in each other’s science through explicit recognition in hiring, performance reviews, resource allocation, and authorship practices of intellectual and experimental contributions to collaborative work.

vii) Forming project teams – groups of dedicated scientists that take research tools or data resources developed in individual labs from proof-of-principle to broadly useful products. For example, the three papers reporting the GCaMP series of calcium sensors have been cited over 3,600 times, and Addgene has distributed copies of over 4,800 GCaMP-containing plasmids deposited by Janelians. Project teams function much like biotech start-ups, guided by a project scientist with input from Janelia group leaders and senior management. The expertise of their staff differs from that of the intended end users of their products. For example, the team that develops fluorescent sensors for neural activity (https://www.janelia.org/project-team/genie) is largely devoted to protein engineering and high-throughput assays. Similarly, the team determining the wiring diagram of the fly brain (https://www.janelia.org/project-team/flyem) develops novel electron microscopy and computational image analysis methods. Janelia’s project teams have had disproportionate impact because the tools and datasets they generate accelerate work in hundreds of laboratories. Similar activities are rare in academia, largely due to limitations imposed by career structures and funding mechanisms.

viii) Insulating Janelia from the dominant academic culture through geographical separation, while mitigating the negative affects of isolation through conference and visitor programs.

### Janelia’s talent development and appointment structures

The group leader position at Janelia was envisioned as a distinct alternative to an academic faculty position. From its inception, Janelia sought to recruit both early career scientists under a term-limited contract and more senior investigators with renewable positions. We knew that Janelia’s operational philosophy, career structure, and chosen research foci might only appeal to a small subset of scientists. But we were pleasantly surprised by the strength of its appeal to that subset.

In the current academic system, assistant professors running their first research groups quickly abandon hands-on research in order to satisfy the demands of grant writing, teaching and lab management. While these other activities are certainly worthy, some scientists find this early and abrupt transition away from hands-on research to be counterproductive. A position at Janelia enables scientists to stay "at the bench" during this critical transition to independence. We note that Janelia has also attracted senior scientists eager to return to the direct conduct of science.

Of the 190 individuals who currently work in the 41 laboratories headed by group leaders, 50% are postdoctoral fellows, 40% are scientific staff and 10% are PhD students. In contrast, project teams and shared scientific resources are made up almost exclusively of scientific staff. While we do not grant degrees, 23 students to date have been granted PhDs from either Cambridge University, the University of Chicago or the Johns Hopkins University based on research done at Janelia. In addition, 12 visiting graduate or medical students have spent a year or more conducting research at Janelia.

Janelia has placed an emphasis on establishing a highly supportive environment for work-life balance for early career scientists. We provide on-site child and infant care (with no wait-list), a range of on-campus housing, and release from grant writing, teaching, and committee work. Highly efficient administrative and operational support serves to minimize other time sinks.

### Lessons learned

We learned that granting tenure is not required to recruit excellent scientists; many of our recruits gave up academic tenure to join us. It was also possible to identify individuals for whom postdoctoral training was not required for success in leading a research effort. However, our experience was that such individuals rarely self-nominate, so it required proactive recruiting to identify them.

We learned that small labs can be disproportionately effective in a supportive, collaborative environment focused on a small number of challenging, shared research goals. We confirmed our founding assumption that the co-localization of tool-builders with those who need those tools would greatly speed up the development of new instruments, reagents, and methods that worked reliably and could be widely distributed. However, we found we often could not adequately scale up these activities within our small lab structure. We therefore created project teams that had a different management structure and mix of staff.

We learned that it is critical to explicitly describe the intended research culture and to only recruit individuals who actively prefer, and commit to, working in such an environment. It was generally not feasible to change an individual’s working style preferences or personality after they joined Janelia. We also learned that without an opposing force provided by management, there is a slow, steady drift toward a more conventional environment increasingly focused on maintaining successful programs and documenting individual achievement at the expense of risk-taking and collaborative, interdisciplinary work. We introduced the scheduled turnover of research areas, described below, as a strategy to counteract this drift.

And has Janelia provided a good return on investment? From the perspective of HHMI, Janelia has been highly successful. It has also provided us with a vehicle to experiment with research environments in a way that has proven to be very informative. We are also confident that Janelia passes the "deletion test" of having done work that would not have been done elsewhere in the same time frame. At the time the decision was made to establish Janelia in 2001, the alternative under discussion was to increase the number of HHMI investigators by 20%, from 300 to 360. Instead, HHMI elected to diversify its approach to achieving scientific advances. Many of Janelia's major achievements have been in areas not generally pursued by HHMI investigators, such as delivering validated tools and taking on larger interdisciplinary, collaborative projects. It will take another decade before the importance of those tools and resources for scientific progress become clear, and even then it will be difficult to judge their impact relative to the types of work generally done in academic labs.

## Looking forward

Based on what we have learned over the past 12 years, we are making three major changes to help Janelia remain a vibrant, distinctive force for scientific progress. First, we are introducing defined 15-year terms for areas of research focus. To allow the scheduled introduction of a new research area every five years, we plan to expand the size of Janelia’s research program to add a third research area. Second, we will choose future research areas by open competition to allow broad input from the scientific community. Third, we will place a greater emphasis on hiring group leaders at a very early career stage to empower the next generation of scientific leaders. These changes are described in more detail below.

### A defined term for research areas

The path of least resistance for Janelia would be simply to seek to maintain our established leadership position in our two original research areas of circuit neuroscience and imaging. But we believe this would not be ambitious enough to justify HHMI’s continued investment in Janelia. Rather, we believe Janelia’s greatest opportunity for future impact will come from supporting the early development of new emerging fields. Therefore, we are seeking to identify high impact research areas that are difficult to pursue in other environments, much as circuit neuroscience and optical imaging were when we selected them in 2004.

We do not claim unusual ability to recognize areas worthy of pursuit. Important research areas are often under-studied simply because they fail to align with major funding agency agendas, they require a tolerance for high-risk projects, or they are not well suited to deliver short-term and translational goals. In other cases, progress in a field may require the kind of highly focused, collaborative and patient effort across disciplines that has proven difficult to carry out in academia. The challenge will not be to identify such research problems, but rather to identify talented, passionate and fearless individuals who are committed to solve them and move to Janelia to do so.

If Janelia’s efforts to provide new tools and concepts in a given research area are successful, then that area should mature and attract increased interest and funding from other sources, to the point where it could now be carried out by HHMI investigators and others in academic environments. Conversely, if we are unable to break through the barriers preventing progress in a field in a reasonable amount of time, then perhaps we should concede defeat and abandon that area. It is not possible to predetermine how long it will take to develop an area of research to the point where Janelia’s involvement has diminishing returns, or even to recognize in real time when such an inflection point has been reached.

Given these uncertainties, we feel it is important to set a defined period of support for new research areas. A defined term ensures turnover, allows Janelia to impact additional fields, and allows us to take advantage of new areas and developments as they come along. It also creates a sense of urgency and common purpose among the participating scientists and motivates effective collaboration across disciplines. We have chosen 15 years for this term length, a time period supported by observing the progress we have made in our current research areas over 12 years. Success of the collective effort means that the participants will be heavily recruited by other institutions as an area nears its conclusion at Janelia. Such transitions are supported by HHMI’s generous transferable research support.

**Figure 2.**
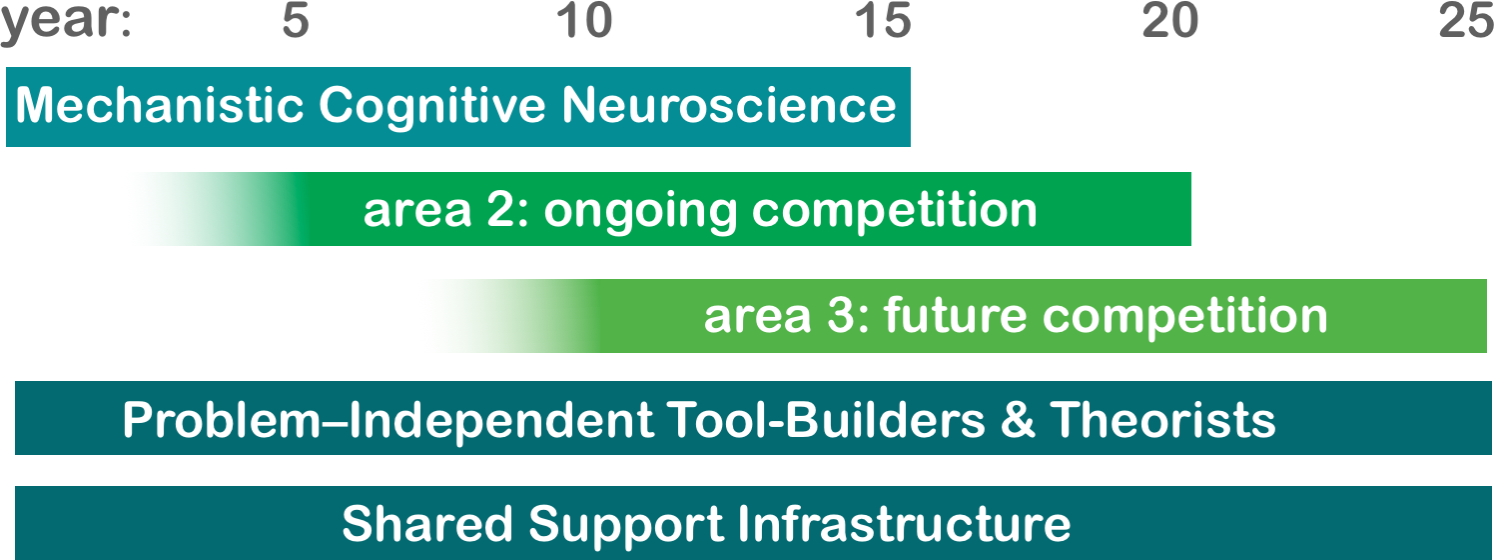
Plan for the scheduled turnover of research areas at Janelia. The first new research area, Mechanistic Cognitive Neuroscience, will address the question "How does the brain enable cognition?" The scientists establishing this area have been selected from current Janelia group leaders working on several model organisms. Additional group leaders are now being recruited. The second research area will be selected after an open, international competition in 2019/2020. We expect a third research area to be selected similarly in 2024/2025. Thus, at steady state, Janelia will have three research areas, with one turning over every five years. The addition of a third research area would represent an expansion of Janelia’s current size and increase the number of group leaders from roughly 45 to 60.

The first of the new areas, Mechanistic Cognitive Neuroscience, is being established by consolidating, refocusing and extending our current neurobiology programs into more cognitive questions (https://www.janelia.org/news/what-mechanistic-cognitive-neuroscience). The other areas will be chosen by open competition; the first of these competitions has been announced (https://www.janelia.org/our-research/competition/opportunity), and we expect a competition to establish a third research focus to follow in approximately five years (Figure 2).

### Modeling an alternative career path for early career scientists

**Figure 3.**
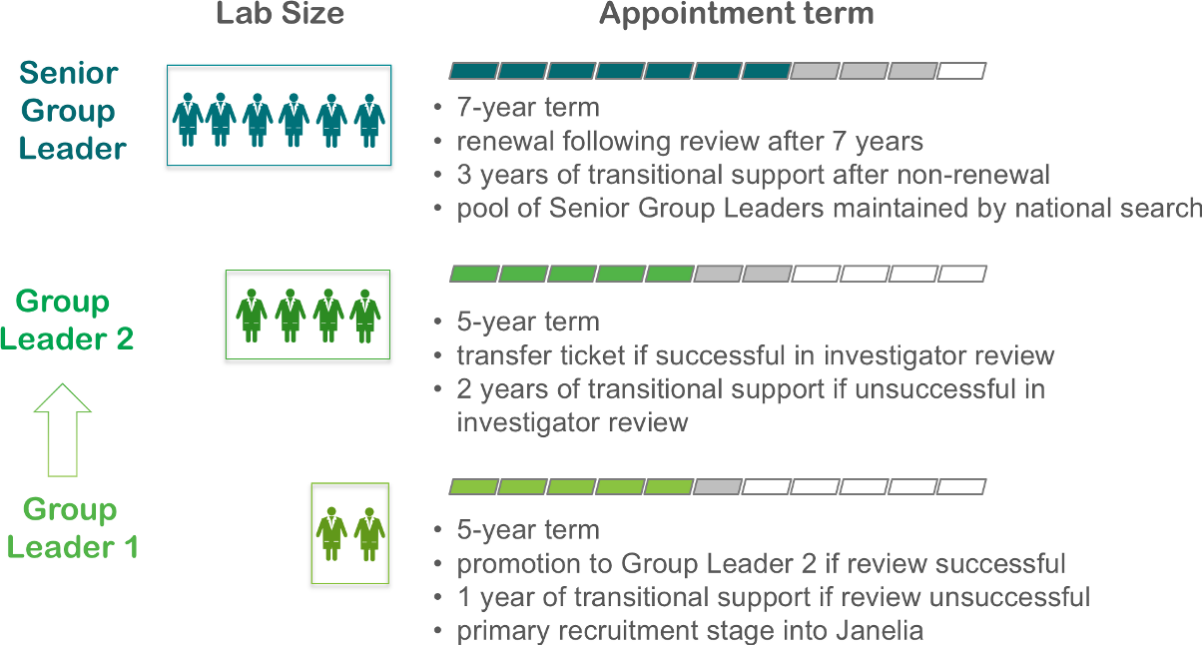
Group leaders at Janelia. Individuals recruited at the start of their independent careers are given an initial five-year term and provided with funds to support two lab members plus access to Janelia’s shared scientific resources. Because the group leader is able to focus on their research, and also has access to the shared infrastructure at Janelia, we believe this group size is equivalent to a group of five in a typical academic setting. Collaboration between groups with different expertise can further increase a group’s intellectual and technical breadth and thus its virtual size. For those who pass their first review, the appointment will be extended for another five years accompanied by an increase in group size. At the end of this second appointment, individuals will be eligible to compete for a "transfer ticket" that would allow them to continue their HHMI support as an HHMI investigator at another institution; those who do not pass their ten-year review will be given two years of transitional funding (roughly corresponding to $1.5 million). Senior group leaders will make up about one-third of all group leaders in each research area and have seven-year terms that can be renewed one time. In addition to running their own research groups, they are expected to contribute to the recruitment and mentoring of their more junior colleagues. Senior group leaders can also compete for a transfer ticket that allows them to move to another institution as an HHMI investigator at the end of their term, and if they are not successful they will be given transitional funding roughly equivalent to three years of their annual budget.

Janelia seeks to catapult the careers of scientists. Going forward, we anticipate placing an even greater emphasis on identifying, recruiting and supporting group leaders who have the intellect, scientific creativity, and maturity to flourish independently without the need for traditional, lengthy postdoctoral training. The terms and conditions of group leader positions are aligned with these objectives (Figure 3). It is our hope that these scientists will provide an inspirational presence at Janelia, shaping the culture and progress of science in unique ways through a combination of confidence, willingness to take risks, and disinterest in traditional disciplinary boundaries. We are encouraged by the success of the 20% of group leaders who were recruited by Janelia directly after graduate training. We are also optimistic that proactively identifying individuals earlier in their careers, before many women and other underrepresented groups have abandoned careers in research, will help us tap into a more diverse applicant pool. In addition, Janelia has hired individuals from industry as well as "brilliant misfits" – scientists with non-traditional backgrounds who would be unlikely to thrive as university faculty – and seen them flourish at Janelia; we will continue to welcome such unusual individuals and provide them with an opportunity to contribute to the basic research enterprise.

There is tension between what is best for an individual scientist’s short-term market value and what is best for science and for the long-term success of an institution that depends on interdisciplinary, collaborative and long-range work. Nearly all Janelia scientists will ultimately take positions elsewhere, so it is only natural that the prevailing practice of attributing credit to individuals based on their position in the author list of publications might limit their engagement in collaborative work. Moreover, many scientists who have helped develop a highly productive research area will want to reap the conventional rewards of continuing in that same area of study rather than abandon it to develop a new area.

To address these issues, Janelia has sought to align individual and institutional goals as much as possible through its hiring practices, internal reward structures, and provision of transitional support to departing group leaders. For individuals considering a position at Janelia, we make our expectations clear that they would supervise a small group, work in the lab themselves, and participate in collaborative, interdisciplinary efforts beyond the scope of an individual laboratory. With this model, we provide a home for those who may not fit well in current academic settings. We then empower their efforts by providing generous internal funding, shared scientific resources and technical expertise, and the ability to work at scale through project teams. Finally, we provide generous transitional funding – which for some individuals can include appointment as an HHMI investigator – that make them attractive recruitment targets for other institutions at the conclusion of their stay at Janelia.

Janelia also provides well-compensated, long-term career research positions for individuals who are not group leaders; however, *no* HHMI position at any level is permanent or tenured. It has been widely noted (see, for example, Alberts et al., 2014) that the number of faculty positions available is insufficient to provide meaningful careers in basic research for the large number of talented individuals in training. Nor do all trainees wish to take on the non-research duties required of principal investigators. The number of such career staff scientist positions at Janelia far exceeds the number of group leaders – three-quarters of those involved in research with six-figure salaries are not group leaders – and these individuals provide important expertise and continuity in our laboratories, project teams and shared resources. Moreover, their career structure rewards them for participating in collaborative, high-risk, and long-term projects that are less suited for graduate students and postdoctoral fellows.

### Continued support for tool-builders and theorists

The other major areas of scientific activity at Janelia are, and will continue to be, tool-building (https://www.janelia.org/news/tools-of-the-trade-q-and-a-with-luke-lavis) and computation and theory (https://www.janelia.org/news/digging-into-data-qa-with-kristin-branson). We expect these activities to evolve on a less defined timeline than the problem-focused research areas. Janelia’s distinctive support of independent, expert tool-builders has allowed the development of broadly useful tools applicable to nearly any research area we might target (new light and electron microscopic imaging technologies; computational methods in machine learning, data analysis, and modeling; and new chemical and biological reagents). For example, the new microscopes, fluorescent dyes, and sensors developed at Janelia are now used in thousands of laboratories worldwide, not only to conduct research in neurobiology, but also in developmental biology, cancer, infectious disease, immunology, and cell biology.

Janelia also recognizes the importance of theory in conceptualizing, prioritizing and designing complex experiments and understanding novel datasets enabled by new experimental methods. As with the shared scientific resources, these tool-building and theory activities will continue to provide research infrastructure and sophisticated technological capabilities for new research topics, adapting and changing as necessary.

## Concluding remarks

Janelia is an ongoing experiment in the conduct of scientific research. As in any other ongoing experiment, we have looked back at what we have learned over the past decade to modify the working hypotheses that will guide us going forward. Encouraged by the successes of those we have hired with little or no postdoctoral training, we plan to increase the proportion of our group leaders who are hired at an early career stage. Motivated by the observation that our greatest impact has come from energizing emerging fields and empowering them through tool development, we have instituted a mechanism that will ensure scheduled turnover of research areas and reaffirmed our commitment to tool development and distribution. Janelia will continue to be a community of small labs that collaborate across disciplines, enriched by project teams, support staff, visiting scientists and scientific conferences. We are optimistic that such a structure will encourage our group leaders to keep working at the bench, take risks, and ambitiously aim for tools and discoveries that could transform their fields.

Changing areas of research focus as science advances is important to maintain the vitality of any research institution. A policy of *scheduled* turnover of research foci, however, is much more radical, and Janelia may be unique among research institutions in having both the flexibility to try this model and the willingness to take on this experiment. Janelia is not tied to any particular research area, having been established with the primary goal of conducting research in a distinct way, rather than conducting research on a particular set of problems. By providing a path for highly successful scientists at Janelia to enter the HHMI Investigator program, we are able to separate judgments about the quality of a scientist from the evaluation of the merit of Janelia continuing research in their area of interest. The fact that we are internally funded gives us the freedom to work in emerging research areas before they are valued by funding agencies. We expect Janelia-trained group leaders, postdocs and other scientists to use their experiences at Janelia to transform their future host institutions and help identify future recruits at all levels for Janelia.

We recognize that there are challenges to accomplishing turnover at this scale in a graceful way, as well as risks in giving up research programs known to be highly productive in order to pursue new ideas. Nevertheless, we are excited by the possibility that always having a new research area in its formative stage, populated by scientists who are dependent upon each other to succeed, will help reestablish the start-up feel of Janelia’s early years. In addition to preparing Janelia for a second decade of experimentation, talent development and exciting science, we believe that the changes we have described here will also help ensure that Janelia continues to play a distinct and valuable role in HHMI’s overall support of science.
